# Current and novel therapeutic opportunities for systemic therapy in biliary cancer

**DOI:** 10.1038/s41416-020-0987-3

**Published:** 2020-07-22

**Authors:** José J. G. Marin, Maria Giuseppina Prete, Angela Lamarca, Simona Tavolari, Ana Landa-Magdalena, Giovanni Brandi, Oreste Segatto, Arndt Vogel, Rocío I. R. Macias, Pedro M. Rodrigues, Adelaida La Casta, Joachim Mertens, Cecilia M. P. Rodrigues, Maite G. Fernandez-Barrena, Ana Da Silva Ruivo, Marco Marzioni, Giulia Mentrasti, Pilar Acedo, Patricia Munoz-Garrido, Vincenzo Cardinale, Jesus M. Banales, Juan W. Valle, John Bridgewater, Chiara Braconi, on behalf of the working group 6 of the COST-action 18122 (Euro-Cholangio-NET) as part of the European Network for the study of Cholangiocarcinoma (ENSCCA)

**Affiliations:** 1 IBSAL, University of Salamanca, Salamanca, Spain; 2 National Institute for the Study of Liver and Gastrointestinal Diseases (CIBERehd), Carlos III National Health Institute, Madrid, ES Spain; 3 Institute of Cancer Sciences, University of Glasgow, Glasgow, UK; 4 Medical Oncology and Hematology Unit, Humanitas Clinical and Research Center - IRCCS -, Rozzano (MI), Italy; 5 Department of Medical Oncology, The Christie NHS Foundation Trust, Manchester, UK; 6 Division of Cancer Sciences, University of Manchester, Manchester, UK; 7 Medical Oncology Unit, S. Orsola-Malpighi University Hospital, Bologna, Italy; 8 Department of Liver and Gastrointestinal Diseases, Biodonostia Health Research Institute, Donostia University Hospital, University of the Basque Country (UPV/EHU), San Sebastian, Spain; 9 Department of Experimental, Diagnostic and Specialty Medicine (DIMES), Alma Mater Studiorum-University of Bologna, Bologna, Italy; 10 Unit of Oncogenomics and Epigenetics, IRCCS Regina Elena National Cancer Institute, Rome, Italy; 11 Department of Gastroenterology, Hepatology, and Endocrinology, Hannover Medical School, Hannover, Germany; 12 Department of Gastroenterology and Hepatology, University Hospital Zurich, Zurich, Switzerland; 13 Research Insitute for Medicines, Faculty of Pharmacy, Universidade de Lisboa, Lisbon, Portugal; 14 CIMA, University of Navarra, IdisNA, Navarra, Spain; 15 Coimbra Hospital and University Centre (CHUC), Coimbra, Portugal; 16 Università Politecnica delle Marche/Ospedali Riuniti di Ancona, Ancona, Italy; 17 Institute for Liver and Digestive Health, University College London, London, UK; 18 Biotech Research & Innovation Centre (BRIC), University of Copenhaghen, Copenhagen, Denmark; 19 La Sapienza University, Rome, Italy; 20 IKERBASQUE, Basque Foundation for Science, Bilbao, Spain; 21 UCL Cancer Institute, London, UK

**Keywords:** Biliary tract cancer, Molecular medicine

## Abstract

Biliary tract cancers (BTCs) are a group of rare and aggressive malignancies that arise in the biliary tree within and outside the liver. Beyond surgical resection, which is beneficial for only a small proportion of patients, current strategies for treating patients with BTCs include chemotherapy, as a single agent or combination regimens, in the adjuvant and palliative setting. Increased characterisation of the molecular landscape of these tumours has facilitated the identification of molecular vulnerabilities, such as *IDH* mutations and *FGFR* fusions, that can be exploited for the treatment of BTC patients. Beyond targeted therapies, active research avenues explore the development of novel therapeutics that target the crosstalk between cancer and stroma, the cellular pathways involved in the regulation of cell death, the chemoresistance phenotype and the dysregulation of RNA. In this review, we discuss the therapeutic opportunities currently available in the management of BTC patients, and explore the strategies that can support the implementation of precision oncology in BTCs, including novel molecular targets, liquid biopsies and patient-derived predictive tools.

## Background

Biliary tract cancers (BTCs) comprise a group of rare and aggressive malignancies that arise in the biliary tree, a complex system of ducts accounting for the modification and transfer of bile from the canaliculi, where it is initially generated, to the duodenum.

BTCs include cholangiocarcinoma (CCA), gallbladder cancer (GBC) and ampulla of Vater cancer (AVC). The studies mentioned in this paper often include a combination of all biliary cancers. More recently, dedicated trials to CCAs without GBCs and AVC are being conducted. Biliary ampullary cancers are rare tumours, and to date, no dedicated trials have been set up, so their management follows the indication of the rest of BTCs.

According to the updated anatomical classification, CCA can be further subdivided into intrahepatic (iCCA), perihilar (pCCA) and distal (dCCA) cholangiocarcinoma, which also reflects differences in epidemiology, aetiology, embryology, biology, prognosis and strategy for clinical management. Based on previous data, CCA has also been classified as iCCA, originating from the biliary tree within the liver, and extrahepatic cholangiocarcinoma (eCCA), which occurs outside the liver parenchyma, and includes perihilar and distal ducts.

Comprehensively, BTCs represent 3% of all gastrointestinal cancers, and are the second most common type of primary liver cancer after hepatocellular carcinoma. Worldwide, the incidence and mortality of BTCs are rising.^1^ Although the incidence is much higher in Eastern countries (up to 85 per 100,000 in Thailand) compared with the rest of the world due to the liver flukes, studies show that CCA rates are rising in most Western countries. In the United States, a country with one of the lowest incidence rates, BTC incidence increased with an annual percentage change of 4.36% in the last decade, reaching a value of 1.6 per 100,000.^2^ Multiple risk factors are known to be associated with BTC development, including liver fluke, biliary tract disorders, chronic liver diseases and metabolic syndrome.^3^

BTCs are characterised by clinical and pathological heterogeneity, showing a poor response to chemotherapy and dismal prognosis. Due to the asymptomatic behaviour of the disease, most of the patients with BTCs are diagnosed at advanced stage. Only patients with localised disease (20%) benefit from surgical resection. However, the recurrence rate is very high, with a median 5-year survival of <50% in resected patients. For patients with advanced unresectable or metastatic BTCs (approximately 60–80%), systemic therapies are the only potential therapeutic options, and the median overall survival (mOS) is poor, ranging from 6 to18 months.^4^

In an attempt to improve the clinical outcome of patients with BTCs, shared efforts are moving towards two goals: the identification of molecular alterations and prognostic factors that can guide treatment, and the development of novel therapeutics and combination strategies. We begin this review by outlining the currently available therapeutic strategies for BTC patients before discussing personalised oncology as an approach for the management of these patients.

## Systemic therapy for cholangiocarcinoma: where do we stand?

### Adjuvant therapy

The incidence of locoregional and distant relapse remains high in patients with resected BTCs. Until 2017, the use of adjuvant treatment was based on meta-analysis data from small and retrospective Phase 2 studies showing an improvement in OS in two high-risk populations: those with node-positive disease and those with R1 resection.^5^ Subsequently, the results of three prospective randomised clinical trials (RCTs) exploring experimental adjuvant chemotherapy arms in resected BTC patients have been published.^6–8^ In the Japanese BCAT trial,^6^ 226 patients with eCCA were randomly assigned to gemcitabine or observation alone following surgery. The study did not meet its primary endpoint, with no significant differences in mOS (62.3 vs. 63.8 months, respectively; hazard ratio [HR] 1.01, 95% confidence interval [CI] 0.70–1.45; *P* = 0.964) or relapse-free survival (RFS; median 36.0 vs. 39.9 months; HR 0.93, 95% CI 0.66–1.32; *P* = 0.693) between the two groups. The French PRODIGE-12/ACCORD-18 study^7^ also failed to show a benefit in response to the adjuvant combination of gemcitabine and oxaliplatin (GEM/OX) compared with observation alone in patients following resection of CCA and GBC; this study did not meet its primary endpoint, with no benefit in terms of RFS in the doublet-chemo arm (30.4 months vs. 18.5 months in observational arm; HR 0.88, 95% CI 0.62–1.25; *P* = 0.48). The BILCAP study,^8^ conducted in the United Kingdom over a period of 9 years, is the largest study so far involving patients with CCA and patients with GBC. Although the study did not meet its primary endpoint in terms of OS in the intention-to-treat population (ITT), the pre-specified ITT sensitivity analysis adjusted for prognostic factors (nodal status, grade of disease and gender) and the per-protocol population analysis did show a longer mOS in the capecitabine arm (53 months vs. 36 months in the observational arm, HR 0.75, 95% CI 0.58–0.97; *P* = 0.028). In the ITT analysis, median RFS was longer with capecitabine (24.4 months, 95% CI 18.6–35.9) compared with observation (17.5 months, 95% CI 12.0–23.8), but no differences in the risk of relapse were demonstrated after 24 months.

As well as differences in BTC subtypes, heterogeneity in the populations enrolled in these three adjuvant trials with regard to node involvement and resection margins should be noted.^9^ The higher proportion of patients with poor prognostic factors could partly explain why the BILCAP trial is the only study that demonstrates a beneficial effect of adjuvant chemotherapy in patients with resected BTCs. Importantly, it should be noted that the three RCTs differ in sample-size calculation, statistical power of study design, maturity of data and follow-up time. Future efforts in designing multicentre, randomised Phase 3 trials should aim to standardise risk factors and include them in pre-planned analyses to obtain a more optimal patient selection and study design. The largest ongoing study evaluating the efficacy of adjuvant therapy in patients with BTC is the ACTICCA study, which compares gemcitabine and cisplatin chemotherapy (GEM/CIS) with capecitabine alone (ClinicalTrials.gov: NCT02170090).

### First-line chemotherapy

GEM/CIS is currently the standard first-line treatment for patients with advanced BTC (aBTC), based on the results of Advanced Biliary Tract Cancer (ABC-02) Phase 3 and the Japanese BT22 Phase 2 trials, which demonstrated the superiority of this combination compared with gemcitabine monotherapy.^10,11^ However, to improve further on the modest survival benefit conferred by GEM/CIS, other first-line chemotherapy options are under investigation. The FUGA-BT trial reported non-inferiority of gemcitabine plus S1 (a fluoropyrimidine derivative) chemotherapy compared with GEM/CIS, suggesting that this treatment could represent another option for aBTC.^12^ Furthermore, a Phase 2 study evaluating nanoliposomal irinotecan in combination with fluorouracil (5-FU)/leucovorin versus GEM/CIS is ongoing.^13^ Beyond doublet therapy, a Phase 2 triplet approach with nanoparticle albumin-bound (nab)-paclitaxel plus GEM/CIS attained the highest mOS (19.2 months) reported in this setting;^14^ this combination is currently under evaluation in a randomised Phase 3 study versus GEM/CIS (S1815 SWOG clinical trial).

A 2020 post hoc analysis of the results from prospective, randomly assigned ABC-01/02/03 trials of GEM/CIS shows a longer OS (by ~4 months) of patients with iCCA compared with non-iCCA-BTC patients and suggests—albeit with a low level of evidence due to the small size—a more favourable prognosis of iCCA and iCCA with liver-only disease.^15^ Such a difference might be of relevance when assessing the suitability of sequential liver-directed therapies on the OS of these patients. Two Phase 2 trials combining gemcitabine and platinum derivatives with concomitant liver-directed therapies (radioembolisation with yttrium-90 [a technique in which microspheres emit β-radiation to block the supply of blood to the tumour] and intra-arterial infusion) yielded interesting median OS figures (22 and 25 months, respectively).^16,17^ Confirmatory Phase 3 studies of radioembolisation are awaited.

When evaluating OS, it is also important to consider the impact of prognostic factors (also relevant for patient stratification). The post hoc analysis of GEM/CIS pivotal trials^10,11,15^ suggests a prognostic role for Eastern Cooperative Oncology Group (ECOG) performance status (PS), white blood cells, haemoglobin, disease status, bilirubin, neutrophil count and gender, but these data have not yet been confirmed.^15^ In a real-life setting, a study conducted by the G.I.Co. (Italian Group of Cholangiocarcinoma) involving 940 Italian patients with aBTC captures ECOG, prior resection, tumour grading, baseline carcinoembryonic antigen and carbohydrate antigen 19.9 as factors that are independently associated with OS.^18^ Further studies incorporating putative molecular prognostic factors, such as the fibroblast growth factor receptor (*FGFR*)-2 fusions, are needed to identify genomic prognostic variables that might help to identify prognosis and predict treatment outcomes.

### Second-line chemotherapy

The benefit of any second-line treatment for patients with BTC has been unclear until the past year. A systematic review published in 2014 showed that studies available in the second-line setting were of limited quality, with 14 out of 25 eligible studies representing Phase 2 clinical trials and no RCTs being identified.^19^ Data from a total of 761 individual patients were reported; the pooled mOS, PFS, response rate (RR) and disease-control rate (DCR) were 7.2 months (95% CI 6.2–8.2), 3.2 months (95% CI 2.7–3.7), 7.7% (95% CI 4.6–10.9) and 49.5% (95% CI 41.4–57.7), respectively. Although the available data suggested that a subpopulation of patients, especially young patients and those with a good PS, could benefit from second-line chemotherapy, this benefit seemed limited, and the evidence was considered to be of insufficient quality (level C) to recommend second-line chemotherapy for aCCA as a standard-of-care strategy.^20^ One of the main challenges for the completion of adequately powered studies was the fact that, due to the aggressive behaviour of CCA, few patients (ranging from 10 to 40% in different series) are considered to be eligible for second-line treatment.^18,21–23^ In 2019, the results from the ABC-06 clinical trial were reported.^24^ This Phase 3 study recruited 162 patients diagnosed with aBTC (72% of whom had a diagnosis of CCA) following progression on first-line GEM/CIS chemotherapy. Patients were randomly assigned to active symptom control (ASC, 81 patients) or ASC with FOLFOX (5-FU and oxaliplatin, 81 patients). The study met its primary endpoint, showing a benefit from second-line chemotherapy in terms of OS (adjusted HR 0.69, 95% CI 0.50–0.97; *P* = 0.031). Even though absolute differences in mOS were modest (5.3 months [ASC arm] vs. 6.2 months [ASC + FOLFOX arm]), differences in the survival rate at 6 months (35.5% [ASC arm] vs. 50.6% [ASC + FOLFOX arm]) and 12 months (11.4% [ASC arm] vs. 25.9% [ASC + FOLFOX arm]) were clinically meaningful. Therefore, FOLFOX is currently being considered as standard-of-care second-line chemotherapy for patients with aBTC previously treated with GEM/CIS.

Novel chemotherapy strategies, such as FOLFIRINOX (5-FU, irinotecan and oxaliplatin)^25^ and etoposide toniribate (EDO-S7.1),^26^ are being tested in the second-line setting, but their efficacy requires confirmation. The Phase 2 studies NALIRICC (ClinicalTrials.gov: NCT03043547) and NAPOLI-2 (ClinicalTrials.gov: NCT04005339) are currently assessing the nanoliposomal irinotecan/5-FU/leucovorin versus 5-FU/leucovorin in patients previously treated with gemcitabine-based therapies.

### Targeted therapies on the horizon

The molecular landscape of BTCs has begun to emerge over the past decade, offering researchers and clinicians the potential to develop novel molecularly targeted therapies (Table 1). Accordingly, molecular profiling of CCA tumours has become increasingly significant over the past few years due to the identification of potentially druggable molecular alterations, such as mutations in *IDH1/2* and *FGFR2* fusions. Mutations in *IDH1/2* disrupt the normal catalytic activity of isocitrate dehydrogenase 1/2, causing the altered protein to produce a new metabolite 2-hydroxyglutarate (2-HG), which induces several oncogenic changes to cellular metabolism. *FGFR2* fusions contain the intact kinase domain fused to a large number of different partners, including *BICC1*, *AHCYL1*, *TACC3*, *MGEA5* and *PPHLN1*,^27^ leading to the constitutive activation of the *FGFR2* fusion protein (FFP) and its consequent downstream oncogenic pathways.^27^ The would-be therapeutic effect of acting on these potentially targetable alterations is currently being evaluated.

**Table 1:** Novel opportunities for targeted therapeutics in biliary cancers: ongoing clinical trials.

Trial number	Phase	Pathways targeted	Drug	Condition	Line of treatment	Primary outcomes	Secondary outcomes
NCT03521219	2	*VEGFR2*	Apatinib	aCCA	II	DCR	ORR, OS and PFS
NCT03427242	2	*VEGFR2*	Apatinib	aBTC	II	PFS	OS
NCT02520141	2	*VEGF*	Ramucirumab	aBTC	II	PFS	CR, PR, SD and OS
NCT03377179	2	*SK2*	Opaganib/ HCQ	aCCA	II	ORR and DDCR	Safety, tolerability, pharmacokinetics, PFS, DCR and OS
NCT03185988	2	*HER2*	Trastuzumab	HER2-positive aBTC	II	CR, PR and SD	DCR, PFS, OS, TTR and DoR
NCT04209465	1/2	*EGFR/ErbB*	BDTX-189	EGFR/ErbB mutant aBTC	II	RP2D and ORR	Safety, tolerability and pharmacokinetics
NCT03833661	2	PD-L1; TGFβ Trap fusion protein	M7824	aBTC	II	ORR	AE, DOR, PFS, OS and pharmacokinetics
NCT02908451	1	*TAA AG7*	AbGn-107	aBTC	II	AE and safety	Pharmacokinetics and pharmacodynamics
NCT02576431	2	*NTRK*	Larotrectinib	NTRK fusion aBTC	II	ORR	PFS, OS and DoR
NCT02568267	2	*NTRK*	Entrectinib	NTRK1/2/3, ROS1 and ALK rearrangement aCCA	II	ORR	PFS, DoR and TTR
NCT03207347	2	*PARP*	Niraparib	BAP1 and other DDR pathway- deficient CCA	II	ORR	PFS, OS and DoR
NCT03422679	1/2	*NOTCH*	CB-103	NOTCH alteration CCA	II	DLT and antitumour efficacy	Pharmacokinetics
NCT03873532	2/3	*MAPK*	Surufatinib	aBTC	II	OS	PFS, DCR and DoR
NCT03149549	1/2	*TAA* CD166	CX-2009	aBTC	II	DLT	ORR

*aBTC* advanced biliary tract cancer, *aCCA* advanced cholangiocarcinoma, *PFS* progression-free survival, *OS* overall survival, *DCR* disease-control rate, *DOR* duration of response, *ORR* overall response rate, *PR* partial response, *CR* complete response, *SD* stable disease, *CBR* clinical benefit rate, *TTR* time to response, *AE* adverse events, *QoL* quality of life, *DDR* DNA-damage response, *DLT* dose-limiting toxicity, *TAA* tumour-associated antigen, *RP2D* recommended Phase 2 dose, *HCQ* hydroxychloroquine sulfate.

In the ClarIDHy Phase 3 trial, 185 patients with *IDH1*-mutant CCA following progression on standard-of-care chemotherapy were randomised to receive the *IDH1* inhibitor ivosidenib or placebo. The primary endpoint was met, with a median PFS of 2.7 versus 1.4 months for patients receiving ivosidenib and for placebo group, respectively (HR 0.37, 95% CI, 0.25–0.54; *P* < 0.001). ITT analysis revealed a mOS of 10.8 months in the experimental group versus 9.7 months in the placebo group.^28^ Ongoing clinical trials are also exploring the efficacy of poly(ADP ribose) polymerase (PARP) inhibitors in *IDH1/2* mutant iCAA (as *IDH1* mutations render tumours sensitive to PARP inhibition) in order to assess their synthetic lethality and to target *IDH1/2*-related dependencies (ClinicalTrials.gov: NCT03212274 and NCT03878095).

Phase 2 clinical trials showed meaningful clinical benefits of *FGFR* inhibitors in the treatment of chemorefractory iCCA patients carrying *FGFR2* fusions, which constitute the most clinically responsive group of patients. In a Phase 2 trial assessing the pan *FGFR* inhibitor BGJ398/infigratinib,^29^ the objective RR (ORR) and DCR were 18.8% and 83.3%, respectively, while another pan *FGFR* inhibitor, ARQ087/derazantinib, resulted in an ORR and DCR of 20.7% and 82.8%, respectively, in a Phase 2 trial.^30^ The FIGHT-202 study tested the *FGFR1–3* inhibitor pemigatinib in 107 patients with *FGFR2* fusions, obtaining an impressive 35.5% ORR, with a median duration of response of 7.5 months and PFS of 6.9 months.^31^ Currently there are several *FGFR* inhibitors that differ with respect to their toxicity and specificity through the target range (*FGFR1–4*) under clinical investigation, including Debio 1347, TAS-120/futibatinib and erdafitinib^29,30,32–35^ (Table 2). Infigratinib, pemigatinib and futibatinib have progressed to Phase 3 evaluation as first-line single agents versus the standard-of-care GEM/CIS (ClinicalTrials.gov: NCT03773302, NCT03656536 and NCT04093362), with the trial results eagerly awaited.^36^

**Table 2:** Inhibitors of *IDH1*/*2* and *FGFR2* fusions: current clinical trials.

Trial number	Phase	Pathways targeted	Drug	Condition	Line of treatment	Primary outcomes	Secondary outcomes
NCT03656536	3	*FGFR*	Pemigatinib	*FGFR2* rearrangement aCCA	I	PFS	ORR, DoR, DCR, AE and QoL
NCT03773302	3	*FGFR*	Infigratinib (BGJ398)	*FGFR2* gene fusion aCCA	I	PFS	OS, ORR, DOR, DCR and AE
NCT04093362	3	*FGFR*	Futibatinib (TAS-120)	*FGFR2* gene arrangement aCCA	I	PFS	ORR, DCR, OS, safety and tolerability
NCT04256980	2	*FGFR*	Pemigatinib	*FGFR2* rearrangement aCCA	II	ORR	PFS, DOR, DCR and OS
NCT03230318	2	*FGFR*	Derazantinib	*FGFR* fusions, mutations and amplifications advanced iCCA	II	PFS at 3 months	EORTC QLQ-C30, OS and DOR
NCT02150967	2	*FGFR*	Infigratinib	*FGFR* alteration aCCA	II	ORR	PFS, OS and DCR
NCT02052778	1/2	*FGFR*	Futibatinib (TAS-120)	*FGFR* aberration CCA	II	ORR	PFS, OS and DCR
NCT04238715	2	*FGFR2*	E7090	*FGFR2* gene fusion aCCA	II	ORR	PFS, DOR, TTR, OS, DCR and CBR
NCT02699606	2	*FGRF*	Erdafitinib	*FGFR* alteration aCCA	II	ORR	PFS, OS and DCR
NCT03684811	1/2	*IDH1*	FT-2102	*IDH1-*R132 mutant iCCA	II	DLT	Pharmacokinetics, pharmacodynamics, AE, PFS, TTP, DOR and OS
NCT03212274	2	*PARP*	Olaparib	*IDH1* or *IDH2* mutant CCA	II	ORR	PFS, OS and DoR
NCT03878095	2	*PARP*	Olaparib Ceralasertib (AZD6738)	*IDH1* and *IDH2* mutant CCA	II	ORR	PFS, OS, DOR and AE

*aCCA* advanced cholangiocarcinoma, *iCCA* intrahepatic cholangiocarcinoma, *PFS* progression-free survival, *OS* overall survival, *DCR* disease-control rate, *DOR* duration of response, *ORR* overall response rate, *PR* partial response, *CR* complete response, *SD* stable disease, *CBR* clinical benefit rate, *TTR* time to response, *AE* adverse events, *QoL* quality of life.

## Novel opportunities for targeted therapeutics in biliary cancer

### Is there more to know about FGFR2-aberrant tumours?

*FGFR2* fusion transcripts generated by chromosomal rearrangements are found in about 10–15% of patients with iCCA.^37^ The efficacy of first-generation tyrosine kinase inhibitors (F-TKIs) in iCCA patients is limited by the emergence of secondary resistance, a major genetic determinant of which is represented by on-target mutations that prevent access of F-TKIs to the *FGFR2* ATP-binding pocket.^38^ Resistance mutations in FFPs are most often polyclonal*.* In vitro experiments delineated a drug-sensitivity profile of individual FFP mutants congruent with clinical data: thus, while some mutations cause cross-resistance among different F-TKIs (e.g. N550K, L618V and K660M mutations reduce binding to both BGJ398 and Debio 1347), others appear to be drug-specific (e.g. M538I impairs binding of Debio 1347, but not BGJ398).^38^ Interestingly, TAS-120 maintains activity against most resistance mutations detected so far in BGJ398-treated patients, but lacks efficacy against the highly prevalent V565F gatekeeper mutation; Debio 1347, on the other hand, loses activity against most resistance mutations, except V565F.^38^

Rapidly evolving polyclonal FFP mutations represent a clinical challenge. Sequential administration of mutant-specific F-TKIs informed by next-generation sequencing analysis of circulating tumour DNA has been advocated, but its benefit appears to be limited, given the emergence of several clones.^38^ An alternative strategy could be to prevent the emergence of resistance mutations by upfront combination therapies that incorporate, in addition to the F-TKI of choice, agents that are capable of targeting dependencies shared by wild-type and TKI-resistant FFPs. FFPs, including those with resistance mutations, are heat-shock protein 90 (HSP90) clients and are therefore stabilised by these chaperones; as such, they undergo swift degradation upon HSP90 inhibition.^39^ Moreover, F-TKIs and HSP90 inhibitors exert synergistic effects against FFP-transformed cells.^39^ Notably, as latest-generation HSP90 inhibitors lack the liver and ocular toxicities that have limited the clinical development of earlier drugs in this class, they might therefore deserve consideration in the iCCA field.^40^ Along this line, an emerging paradigm postulates that therapeutic targeting of a driver kinase is more efficacious when combined with the blockade of downstream pathway components.^41^

### Other actionable alterations in CCA

With the advent of improved technologies, it has become apparent that there are multiple potentially actionable alterations in BTCs. In addition to *FGFR2* fusions and *IDH1* mutations, many other alterations, such as amplification of the receptor tyrosine kinase *c-MET*, targetable with savolitinib,^42^ and overexpression of the epidermal growth factor receptor (*EGFR*),^43^ require clinical evaluation, although this will always be challenging because of the low number of patients with these changes. Other important events that require further investigation include activation of the Janus kinase/signal transducer and activator of transcription (JAK/STAT) signalling pathway through constitutive activation of STAT3, which is estimated to occur in 58–77% of patients with iCCA (depending on inflammation or proliferation biological class, respectively),^44^ and gain-of-function mutations in protein tyrosine phosphatase non-receptor type 3 (*PTPN3*), which have been reported in ~ 41% of patients.^45^ Moreover, it remains to be seen whether therapeutically inhibiting additional promising targets, such as *HER2*,^46^ *BRAF*^47^ and *BRCA*,^48^ confers a similar benefit to that observed in more common cancers, such as breast (*HER2*), melanoma (*BRAF*) and ovarian malignancies (*BRCA*). Preliminary data from patients with *HER2*-positive aBTC have shown that dual HER2-targeted treatment with pertuzumab and trastuzumab has activity in this setting.^49^ The combination of BRAF and mitogen-activated protein kinase kinase (MEK) inhibitors was also tested in a Phase 1 trial, and showed promising results for CCA patients with the activating *BRAF* V600E mutation.^47^

Nevertheless, there remains a large cohort (∼50%) of patients with no currently actionable alteration. For instance, some of the most frequent genetic mutations in CCA comprise the proto-oncogene *KRAS* and the tumour suppressor *TP53*, for which the options are limited (Table 3). To date, despite the large number of potential therapeutic targets identified by molecular profiling, more advanced genomic technologies might be required to reveal novel actionable alterations in these difficult-to-treat cancers.

**Table 3:** Targetable mutations in CCA.

Molecular alteration	Incidence		Primary tumour site	Possible agents	ESCAT
‘Established’ targets and drugs	*FGFR2* fusion	10%	iCCA	Futibatinib (TAS-120),^38^ Derazantinib (ARQ087),^30^ Infigratinib (BJG398),^36^ Erdafitinib^33^ and Pemigatinib^31^	II-B
	*IDH1* mutation	10%	iCCA	Ivosidenib^28^ FT-1202	I-B
	*HER2* amplification	10%	eCCA/GBC	Pertuzumab–Trastuzumab^46^	–
	*HER*2 mutation	5%	eCCA/GBC	Neratinib–Trastuzumab^46^ Pertuzumab–Trastuzumab^46^	III-A
	MSI-H	1–3%	eCCA/iCCA/GBC	Durvalumab Pembrolizumab	II-B
	*BRAF* V600E mutation	3%	eCCA/iCCA/GBC	Dabrafenib–trametinib^47^	III-A
	*BRCA2* mutation	3%	eCCA/iCCA/GBC	Olaparib^48^	III-A
	*EGRF* mutation/amplification	3%	eCCA/iCCA/GBC	Osimertinib	III-A
‘Experimental’ targets and drugs	*BRAF* non-V600E mutation	1.5%	eCCA/iCCA/GBC	Encorafenib–Binimetinib	IV-A
	*c-MET* amplification	3%	eCCA/iCCA/GBC	Savolitinib^42^	IV-A
	*BAP1*/BRCAness DDR alterations (SMARCA4; ARID1A)	10%	eCCA/iCCA/GBC	Olaparib	IV-A
	*EGFR* amplification	<5%	eCCA/iCCA/GBC	Osimertinib^43^	IV-A
	*NTRK* fusions/*ROS1*	< 5%	eCCA/iCCA/GBC	Larotrectinib–Entrectinib	IV-A
	*PIK3CA* mutation	< 10%	eCCA/iCCA/GBC	Everolimus–Sirolimus	IV-A

*GBC* gallbladder cancer, *iCCA* intrahepatic cholangiocarcinoma, *eCCA* extrahepatic cholangiocarcinoma, *ESCAT* ESMO Scale for Clinical Actionability of molecular Targets, *DDR* DNA-damage repair, *MSI-H* microsatellite instability-high.

Mutations in DNA-damage repair (DDR) genes are present in about 20% of BTCs, especially in extrahepatic BTCs. In these tumours, PARP inhibitors may have a therapeutic role as they counteract the activity of the PARP enzyme to repair single-strand DNA breaks. However, the benefit of olaparib monotherapy has been limited in other gastrointestinal cancers; thus, it is likely that combination treatments will be explored in BTC. PARP inhibitors may be combined with immunotherapy (see below), with anti-angiogenic therapies (given that hypoxia can reduce DDR) or phosphoinositide 3-kinase (PI3K)/MEK inhibitors (that are overactivated in BTC and have been associated with secondary resistance to PARP inhibition).

Epigenetic alterations have also been described in BTCs.^50^ Treatments aimed at reversing these changes have been studied and shown to be promising, such as the histone deacetylase (HDAC) inhibitor resminostat in pretreated BTC patients.^51^

### Immunotherapy: only for the few?

In contrast to the promising data observed with targeted therapies in molecularly defined patients, immunotherapy (given as a monotherapy) has so far been disappointing in patients with anatomically and molecularly uncharacterised aBTC. One of the largest published immunotherapy studies ever is the KEYNOTE-158 Phase 2 clinical trial, which assessed the efficacy of pembrolizumab, an antibody that targets the immune-checkpoint protein programmed death-1 (PD-1), in patients with previously treated solid tumours, including those of the biliary tract. The subgroup analysis of 104 patients with aBTC treated with pembrolizumab revealed an RR of 5.8% with a median PFS of 2 months and a mOS of 9.1 months, regardless of PD-L1 positivity (membranous PD-L1 expression in ≥1% of tumours and associated inflammatory cells or positive staining in stroma).^52^ Consistent with other studies, pembrolizumab showed durable antitumour activity among the few responsive patients.

So far, a high degree of microsatellite instability (MSI-High [H]), occurring in 1–3% of CCA patients (with germline mutations in mismatch repair genes), is the only marker that appears to be predictive of clinical response to immunotherapy. The KEYNOTE-158 study evaluating pembrolizumab in previously treated patients with advanced non-colorectal MSI-H/deficient mismatch repair (dMMR) cancer showed an ORR of 40.9%, median PFS of 4.2 months and mOS of 24.3 months in the BTC cohort of 22 patients,^53^ demonstrating a clinical benefit of pembrolizumab among these patients, consistent with results from other patients with previously treated MSI-H/dMMR non-colorectal cancer assessed in the study.

In order to increase the efficacy of immunotherapy in BTCs, different therapeutic combinations are currently being tested (Table 4). One approach includes the combination of immunotherapy and chemotherapy. Early clinical data from the combination of nivolumab with GEM/CIS as a first-line treatment showed signs of antitumour activity, with an RR of 37%, a median PFS of 4.2 months and mOS of 15.4 months.^54^ This concept of immunotherapy–chemotherapy combination is currently further evaluated in Phase 3 studies. such as TOPAZ-1 and KEYNOTE-966, in which patients are being treated with GEM/CIS alone or with durvalumab (which targets PD-L1, the PD-1 ligand) or pembrolizumab, respectively.

**Table 4:** Immunotherapy combinations: ongoing clinical trials.

Trial number	Phase	Pathways targeted	Drug	Indication	Line of treatment	Primary outcomes	Secondary outcomes
NCT04027764	2	PD-1, chemotherapy	Toripalimab/S1/Albumin Paclitaxel	aBTC	I	ORR	PFS, DCR and OS
NCT03796429	2	PD-1, chemotherapy	Toripalimab/Gemcitabine-S1	aBTC	I	PFS and OS	ORR and safety
NCT04191343	2	PD-1, chemotherapy	Toripalimab/GEMOX	aBTC	I	ORR	NA
NCT03486678	2	PD-1, chemotherapy	SHR-1210/GEMOX	aBTC	I	PFS and AEs	ORR and OS
NCT03111732	2	PD-1, chemotherapy	Pembrolizumab/capecitabine/oxaliplatin	aBTC	II	5-month survival	ORR, OS, safety and tolerability
NCT03785873	2	PD-L1, chemotherapy	Nivolumab/Nal-irinotecan/5-FU, leucovorin	aBTC	II	Phase 1b: DLT; Phase 2: PFS	AE, ORR and OS
NCT04004234	1/2	PD-1, chemotherapy	Manganese/anti-PD-1/nab-paclitaxel–gemcitabine	aBTC	I/II	AE and PFS	DCR, ORR and OS
NCT03478488	3	PDL-1, chemotherapy	KN035/GEMOX	aBTC	I	OS	PFS and ORR
NCT04003636	3	PD-1, chemotherapy	Pembrolizumab/GEM/CIS	aBTC	I	PFS and OS	ORR, DOR and AE
NCT03486678	2	PD-1, chemotherapy	SHR-1210/GEMOX	aBTC	I	PFS	ORR
NCT03875235	3	PDL-1, chemotherapy	Durvalumab or placebo and GEM/CIS	aBTC	I	OS	PFS, ORR and DoR
NCT03046862	2	PDL-1, CTLA-4, chemotherapy	Durvalumab/Tremelimumab/GEM/CIS	aBTC	I	RR	DCR, PFS, DoR and OS
NCT02834013	2	PD-1 and CTLA-4	Nivolumab/Ipilimumab	aBTC	II	ORR	Safety, OS, PFS and DCR
NCT03849469	1	PD-1, CTLA-4 and LAG-3	XmAb^®^22841/Pembrolizumab	iCCA	II	Safety and tolerability	NA
NCT03092895	2	PD-1, VEGF, chemotherapy	SHR-1210/Apatinib, GEMOX or FOLFOX	aBTC	I/II	Safety and tolerability	OS, PFS, DCR, and DoR
NCT04211168	2	PD-1, VEGF	Toripalimab/Lenvatinib	aBTC	II	ORR and AEs	OS, PFS, OS and CBR
NCT03797326	2	PD-1, VEGF	Pembrolizumab/Lenvatinib	Pretreated solid tumours	II	ORR and AEs	DCR, PFS and OS
NCT03895970	2	PD-1, VEGF	Pembrolizumab/Lenvatinib	aBTC	II	ORR, DCR and PFS	OS and DoR
NCT04066491	2/3	PD-L1; TGFβ trap fusion protein, chemotherapy	GEM/CIS with or without Bintrafusp Alfa (M7824)	aBTC	I	DLT and OS	DoR, AE and pharmacokinetics
NCT03937895	1/2	PD-1, SMT-NK	Pembrolizumab/allogeneic NK cell	aBTC	II	DLT and ORR	TTP and toxicity
NCT04057365	2	PD-1, DKK1	Nivolumab/DKN-01	aBTC	II	ORR (CR and PR)	PFS and OS
NCT03250273	2	PD-1, histone deacetylase inhibitor	Nivolumab/Entinostat	aBTC	II	ORR	PFS, OS and DoR
NCT03639935	2	PD-1, PARP	Nivolumab/Rucaparib	aBTC	II	4-month survival and response	CR, PR, SD, PFS and OS
NCT03475953	1/2	PD-1, MAPK	Avelumab/Regorafenib	Metastatic solid tumours	I/II	RP2D and antitumour activity	MTD, DLT and toxicity
NCT03257761	1	PDL-1, DNMT inhibitor	Durvalumab/Guadecitabine (SGI-110)	aCCA	II	DLT and ORR	Safety and tolerability, PFS and OS
NCT03475953	1/2	PD-1, MAPK	Avelumab/Regorafenib	Metastatic solid tumours	I/II	RP2D and antitumour activity	MTD, DLT and toxicity

Combinations of immunotherapy with chemotherapy and other agents.

*PFS* progression-free survival, *OS* overall survival, *DCR* disease-control rate, *ORR* overall response rate, *PR* partial response, *CR* complete response, *SD* stable disease, *DOR* duration of response, *CBR* clinical benefit rate, *DLT* dose-limiting toxicities, *MTD* maximum tolerated dose, *AE* adverse events, *PARP* poly-ADP ribose polymerase, *GEMOX* gemcitabine + oxaliplatin, *GEM/CIS* gemcitabine + cisplatin, *FOLFOX* fluorouracil + folinic acid + oxaliplatin.

The use of immunotherapy together with anti-angiogenic agents has shown high efficacy against hepatocellular carcinoma, but has not so far been successful in the treatment of BTC. In one study, pembrolizumab plus ramucirumab, which inhibits vascular endothelial growth factor (VEGF)-induced angiogenesis, showed limited efficacy in patients with previously treated advanced/metastatic BTC (only 4% in 26 patients), with a mOS of 6.4 months and median PFS of 1.6 months.^55^ Similar to VEGF signalling, targeting the transforming growth factor β (TGF-β) pathway has been shown to promote tumour immunosuppression and, based on encouraging efficacy observed in a Phase 1 study, M7824, a first-in-class bifunctional fusion protein comprising two extracellular domains of TGF-βRII (a TGF-β ‘trap') fused to a human IgG1 monoclonal antibody against PD-L1, is currently being evaluated in combination with GEM/CIS as a first-line therapy for BTC (clinical trial.gov: NCT04066491). Moreover, the immunogenicity resulted from the increased mutational burden (and thus the neoantigenes) caused by the mechanism of action of PARP inhibitors has provided the rationale to assess them with immunotherapy (clinical trial.gov: NCT03639935).

## Potential opportunities to reverse chemoresistance in biliary cancers

### The molecular mechanisms of chemoresistance

**Fig. 1:**
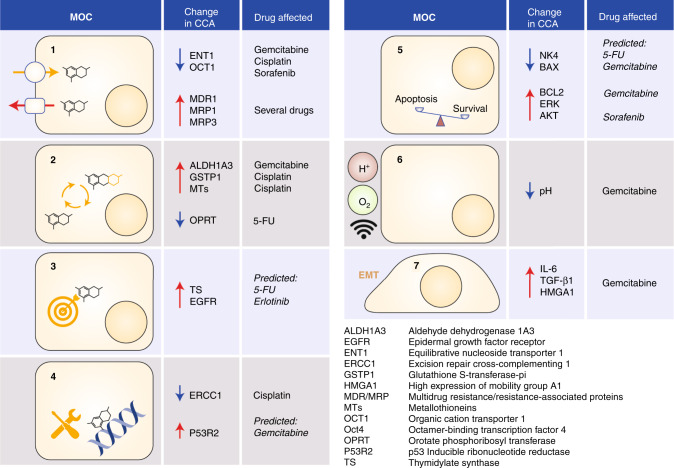
Schematic representation of the molecular mechanisms of chemoresistance, of which there are seven depicted.^56,57^ (1) Changes in the expression/function of transport proteins involved in drug uptake or efflux. (2) A reduction in the intracellular amount of active drugs due to changes in enzymes involved in metabolism. (3) Changes in the molecular targets of anticancer agents. (4) An increased ability of tumour cells to repair drug-induced DNA damage. (5) Decreased expression/function of pro-apoptotic factors or enhanced expression/function of anti-apoptotic proteins. (6) Changes in tumour-cell microenvironment conditions that affect the effectiveness of drugs. (7) Induction of epithelial–mesenchymal transition (EMT).

The mechanisms of chemoresistance to anticancer drugs,^56,57^ which are classified into seven groups (Fig. 1), can already be present in tumours before the start of treatment (primary resistance), although they usually arise in response to the pharmacological challenge (secondary resistance). Drug resistance occurs due to changes in the expression levels or/and the appearance of genetic variants in genes encoding mechanisms of chemoresistance.

Several transporters play a role in resistance by influencing the bioavailability of drugs, both positively and negatively. For instance, upregulation of the human equilibrative nucleoside transporter 1 (hENT1) in CCA cells is associated with a better response to gemcitabine in patients with resected CCA^58^ and aBTC,^59^ and impaired expression of the organic cation transporter 1 (OCT1) is thought to mediate the poor response to cisplatin and the multikinase inhibitor sorafenib.^60,61^ By contrast, ATP-binding cassette proteins, such as MDR1, MRP1 and MRP3, which are highly expressed in CCA, are able to export a wide variety of antitumour drugs out of cells, thereby limiting their effect. Interestingly, high *MRP1* mRNA levels correlate inversely with OS after the treatment of iCCA.^62^ Chemical modification of some conventional chemotherapy drugs has enabled these compounds to enter the cancer cell independently of the above-mentioned membrane transporters, and may represent a good strategy to overcome chemoresistance (clinicaltrials.gov: NCT04163900).

Regarding detoxifying enzymes, the high expression of aldehyde dehydrogenase 1 family, member A3 (ALDH1A3) correlates with a lower response to gemcitabine-based therapy in patients with advanced iCCA,^63^ and glutathione S-transferase-pi (GSTP1), also frequently overexpressed in CCA, has similarly been associated with resistance to cisplatin and other alkylating agents.^64^ Downregulation of metallothioneins is accompanied by a better response to cisplatin.^65^ Other components involved in the mechanisms of chemoresistance include orotate phosphoribosyl transferase (OPRT), a key enzyme in the activation pathway of 5-FU;^66^ accordingly, increased expression of OPRT confers increased sensitivity to 5-FU. By contrast, increased expression of thymidylate synthase (TS), which is involved in DNA synthesis and normally inhibited by 5-FU metabolites, results in lower sensitivity to 5-FU.^67^

In terms of apoptosis/survival genes, CCA resistance to the EGFR inhibitor erlotinib has been associated with the upregulation of EGFR in a feedback loop.^68^ Moreover, increased expression of the p53-inducible ribonucleotide reductase (*p53R2*) gene, which is required for normal DNA repair, correlates with, and has been used to predict, gemcitabine resistance.^69^ Downregulation of the pro-apoptotic protein NK4, an antagonist of hepatocyte growth factor (HGF), is responsible for acquired resistance to 5-FU in CCA,^70^ and downregulation of Bax and upregulation of Bcl-2 contribute to evasion of apoptosis in CCA cells resistant to gemcitabine.^71^ Furthermore, overexpression of anti-apoptotic proteins, such as extracellular signal-regulated kinase (ERK) and Bcl-2, and overactivation of PI3K/AKT and RAF/MEK/ERK pathways, have been identified to be associated with CCA chemoresistance.

Changes in the tumour microenvironment, such as hypoxia, extracellular fluid acidification and the presence of autocrine and paracrine signals, also affect chemoresistance. Upregulation of the octamer-binding transcription factor 4 (Oct4) in acidic conditions has been shown to be associated with CCA resistance to gemcitabine.^72^ Furthermore, the expression of interleukin (IL)-6 and TGF-β1 through an autocrine loop involving Smad4 has been involved in the resistance to gemcitabine by inducing epithelial–mesenchymal transition (EMT).^73^ Moreover, high expression of the mobility group A1 (HMGA1) protein, which promotes EMT, also confers resistance to gemcitabine.^74^ In conclusion, although there continues to be an urgent need to advance our understanding of the mechanisms of chemoresistance, the situation in CCA is starting to be clarified, and novel targets that mediate the contribution of tumour microenvironment in chemoresistance started to be identified for the development of therapeutics that could be clinically investigated.

### MicroRNAs as mediators of chemoresistance and potential RNA therapeutics

MicroRNAs (miRNAs or miRs) are single-stranded non-coding RNAs (18–24 nucleotides) that function as post-transcriptional master regulators to modulate the expression of many genes.^75^ Altered miRNA profiles have been described in many tissues and cells under pathological circumstances, including in CCA,^75,76^ and many miRNAs have been implicated in chemoresistance in CCA patients. For instance, miR-21 is highly expressed in CCA cells compared with non-malignant cells, and its experimental inhibition sensitised cells to gemcitabine through the inhibition of phosphatase and tensin homologue (*PTEN*) in vitro and in vivo,^77^ resulting in decreased PI3K signalling.

Downregulation of miR-200b/c has been reported in CCA, and its enforced expression restores 5-FU sensitivity in CCA cells.^78^ Similarly, miR-29b, miR-205 and miR-221 are downregulated in gemcitabine-resistant CCA cells, but their experimental overexpression restores gemcitabine sensitivity.^79^ The levels of miR-320, which targets the anti-apoptotic protein myeloid cell leukaemia 1 (*MCL1*) and contributes to 5-FU resistance, are diminished in iCCA.^80^ Levels of miR-106b are reduced in 5-FU-resistant CCA cells, but the experimental overexpression of this miRNA re-sensitises them to 5-FU, mainly through the modulation of *Zbtb7a*, a proto-oncogenic transcription factor.^81^ miR-130a-3p levels mediate resistance to gemcitabine by targeting the expression of another transcription factor, peroxisome proliferator-activated receptor (*PPARG*).^82^ Experimental overexpression of *OCT1* in eCCA and iCCA cells enhanced both the uptake and cytotoxic effects of sorafenib. Notably, miR-141 and miR-330 have been shown to target *OCT1*, but the relevance of the modulation of these miRNAs to sorafenib resistance remains to be unveiled.^61^ Functional high-throughput approaches combined with analyses of human tissues have identified miR-1249 as a driver of the expansion of the CD133^+^ subpopulation that is responsible for primary and secondary resistance of CCA cells to cisplatin and gemcitabine.^76^

As the next step for all these findings, it is imperative to evaluate the relevance of these miRNAs in vivo and to correlate their levels with resistance to therapy in patients. Although miRNA-based therapies are already under development, much work needs to be performed in the next few years to improve strategies to synthesise artificial miRNAs and miRNA inhibitors for clinical implementation. It is pivotal to develop and improve new delivery techniques that might help to achieve the best therapeutic efficacy while minimising potential toxic effects.

### Targeting death to improve life

Regulated cell death pathways are central in chronic liver disease progression, where the lack of a balance between cell death and regeneration has been shown to lead to carcinogenesis. Failure of regulated cell death in hepatocytes and cholangiocytes is a pivotal step in malignant transformation. This unique relationship between cell death and liver cancer reflects the importance of chronic damage and inflammation, with the release of several mediators that have oncogenic effects. The balance between different types of regulated cell death might influence the type of liver cancer that eventually develops. For instance, a necroptotic microenvironment with high cytokine levels can promote cholangiocarcinogenesis by activating specific oncogenes, while an apoptotic environment appears to increase the risk of hepatocellular carcinogenesis.^83^ Moreover, a dysregulated equilibrium between anti-apoptotic and pro-apoptotic signals with evasion of both intrinsic and extrinsic apoptosis is a key contributor to the resistance of liver cancer to antitumour drugs, especially in patients with CCA.^84^ The apoptotic mitochondrial pathway is suppressed by overexpression of anti-apoptotic Bcl-2 family proteins, such as Bcl-2^85^ or Mcl-1^86^ in conjunction with downregulation of pro-apoptotic Bcl-2 proteins like Bax.^87^ Similarly, impaired caspase activation caused by overexpression of inhibitors of apoptosis proteins (IAPs) such as XIAP^88^ or survivin,^60^ or abnormal function of death receptors such as Fas (CD95) and DR4/DR5, contributes to the chemoresistant phenotype in CCA cells.

These mechanisms are also regulated by the surrounding microenvironment.^84^ Indeed, cancer-associated fibroblasts (CAFs) are key cells that support the growth of liver tumours, and are sensitised to apoptotic cell death in a characteristic state termed ‘apoptotic priming’.^89^ Pro-apoptotic compounds, such as BH3 mimetics, are being used to exploit this apoptotic priming with encouraging results, reducing tumour growth and metastasis in experimental CCA.^89^ Finally, activation of necroptosis also seems to play a relevant role in CCA by sensitising cells to standard chemotherapy, suggesting novel necroptosis-based therapeutic strategies for CCA patients. Exploring all these different mechanisms of regulated cell death will not only help to understand the powerful mechanisms of chemoresistance, but might also reveal novel opportunities for therapeutic intervention.

### Targeting the interaction with the microenvironment

CCA is characterised by marked abundance of tumour stroma, a bioactive connective tissue that not only physically negatively influences drug delivery, but also crosstalks with cancer cells for the activation of a chemoresistant phenotype.^90^ The CCA stroma consists of cancer-associated endothelial cells, CAFs and inflammatory cells—including tumour-associated macrophages (TAMs), neutrophils, natural killer (NK) and T cells—dispersed in a bioactive specialised extracellular matrix (ECM).^91^ CAFs are mainly responsible for mediating the composition of the ECM and crosstalk with CCA cells by secreting paracrine factors, such as TGF-β and platelet-derived growth factor (PDGF). Among CCA-infiltrating immune cells, TAMs exert a pivotal role in cancer-related inflammation by promoting tumour-cell proliferation, angiogenesis, matrix turnover and suppression of the adaptive immune response. M2-polarised TAMs communicate in particular with chemoresistant CCA cancer stem cells by releasing numerous soluble mediators, including reactive nitrogen intermediates, cytokines (IL-4, IL-6 and IL-10), chemokines (chemokine ligand [CCL] 17 and CCL18) and metalloproteinases (matrix metalloprotease [MMP] 9). Together, TAMs and CCA cells create a tumoral niche that constitutes a potential target for therapy. Following the release of CCL2 by tumour cells and TAMs, cytotoxic T lymphocytes acquire CD4/CD25 expression and become immunosuppressive regulators (Treg cells).^92^ By producing TGF-β and IL-10, Treg cells contribute to an immunosuppressive environment through the inhibition of cytotoxic T cells and NK cells. Moreover, by selective binding, Treg cells make IL-2 inaccessible, thus inhibiting the activation of additional immune cells.^92^ Enrichment of Treg cells has also been associated with chemoresistance in BTC.^93^

As well as cells in the tumour microenvironment, there are other microenvironmental factors linked to the specialised biomatrix components that can significantly impact the behaviour of cancer cells, such as hypoxia, exosomes, proliferative factors and inflammatory cytokines (TGF-β and VEGF).^91^ All these factors play different roles in CCA progression, and might be considered as potential targets for therapy. Nevertheless, exploring the dynamics of immunosuppressive cell subpopulations and their interactions with and within the tumour microenvironment will be essential for a better understanding of drug resistance and the subsequent design of novel strategies for innovative anti-CCA therapies.

## Novel therapeutic strategies for personalised medicine

### Personalised oncology in BTC

Over the past decade, genomic sequencing technologies have helped to shed light on the molecular landscape of BTCs.^37,94^ However, despite the remarkable steps taken to unravel the molecular complexity of this heterogenous disease, the emerging knowledge has only partly been translated into improved clinical management, and hence further studies are needed.

Retracing the path to precision oncology, Verlingue et al.^94^ have demonstrated a tumour-centric approach based on high-throughput genomic analysis of DNA extracted from tumour biopsy samples, selecting potential druggable alterations to match the available target treatments in previously treated BTCs. The prospective MOSCATO-01 trial was successful in determining an outcome improvement (mOS and PFS) in this cohort compared with patients not oriented to molecular targeted agents.^94^ Although preliminary, these results, together with the high frequency of *IDH1/2* and *FGFR2* genetic aberrations confirmed in the trial, have laid the foundation for further investigations. However, as a number of additional targetable molecular alterations have been identified, there is an increasing need to implement our current genetic profiling technologies in clinical practice in order to tailor therapy more appropriately in patients with multiple driver aberrations.

With this information in mind, in the I-PREDICT prospective study, Sicklick et al.^95^ explored the safety and feasibility of a multidrug combination treatment based on a matching score system combining actionable molecular alterations with a corresponding available target therapy or therapies. The most represented population in the study was gastrointestinal refractory tumours (42.2%), including aBTCs. In this study, the ‘matching score’ rate was higher than in previous studies, with 49% of patients receiving multidrug regimens. The highest matching score rate was associated with significantly improved disease-control rates, as well as longer PFS and OS rates, compared with patients receiving therapy matched to fewer genomic alterations.^95^ Therefore, the current clinical trial paradigm, focused on finding common genomic alterations in patients and targeting them with a single agent, might need to be revised in favour of more tailored combination therapies for specific genetic alterations.

### Novel strategies to implement individualisation of treatment: liquid biopsies and patient-derived models

Up to 50% of BTCs are expected to be eligible for targeted therapies, and it has therefore been suggested that genomic profiling is incorporated into routine clinical practice. One of the limiting issues for implementing personalised oncology in BTCs is the lack of tissue for molecular analyses, especially for those BTCs that are diagnosed through cytological sampling. However, this issue might be overcome by the use of liquid biopsies. Mody et al.^96^ presented their experience with a targeted next-generation sequencing panel of 73 genes from the plasma of >120 patients with aBTC. The assessment of molecular alterations was feasible in cell-free DNA (cfDNA), and identification of therapeutically relevant alterations was also successful (*BRAF* and *IDH1/2* mutations, *ERBB2* amplification and *FGFR2* fusions). The limitation of this study was the preponderance of iCCA cases in this cohort, for which lack of tissue is not usually a problem.^96^ Preliminary evidence from only ten patients has demonstrated the possibility of using bile as a source for deep DNA sequencing, showing that cfDNA in bile consists of longer fragments than cfDNA in plasma (with potential higher quality of DNA sequencing), and that there is high correspondence between the mutational profile in bile and BTC tissue.^97^ Further studies are warranted to assess whether bile might be a suitable source of cfDNA for use in the implementation of personalised oncology in patients with advanced pCCA and dCCA. Circulating tumour cells (CTC) are an alternative approach, but to date, low levels of CTC have been detected in BTC limiting their clinical applicability.^98^

DNA sequencing can support precision oncology by identifying targetable molecular alterations. However, it is of no help for guiding treatment decisions in the case of drugs for which predictive biomarkers have not been identified, such as chemotherapy compounds or multityrosine kinases. Patient-derived xenografts (PDXs) have been used for this purpose, but their clinical applicability may be limited by costs and timeframe. Patient-derived organoids (PDOs) are ex vivo, organ-like, three-dimensional structures derived from individual patient cells that could be used to predict response to compounds independently on the presence of a molecular biomarker. Notably, cancer PDOs mimic the structure and genomic heterogeneity of their host tumours, and have been demonstrated to mimic in a dish the drug response observed in patients,^99^ generating excitement on the potential use of these PDOs as predictive tools. Growing evidence is supporting the feasibility of establishing biliary cancer PDOs.^100^ However, the success rate for generating PDOs from different subtypes of biliary cancer is not yet clear, and so more studies are warranted before this approach can be used to support individualised oncology in patients with BTCs. The next few key steps to validate and promote the use of organoids as clinically relevant tools for the study of biliary cancers include the generation of characterised models representing the different CCA subtypes (intrahepatic, perihilar and distal) and the establishment of a collaborative organoid biobank.

## Conclusions

The current guidelines indicate the use of first-line chemotherapy with cisplatin and gemcitabine in aBTC, followed by FOLFOX chemotherapy. Novel targeted therapies (*IDH* and *FGFR* inhibitors) are being considered for iCCA with selected molecular alterations. An ever-increasing number of molecular alterations is being identified, with different BTC subtypes showing specific molecular profiles. Beyond the role of standard chemotherapy, this approach paves the way to design molecular-orientated clinical trials in which different BTC subtypes can be matched to different targeted inhibitors. One common difficulty encountered when studying rare diseases is the low number of cases that can be investigated in a single institution, and this was indeed the case for BTCs until international CCA-dedicated associations were established, with contributions from both basic and clinical researchers in an attempt to join efforts, skills, information and biological samples to improve research in CCA. Although the situation regarding the available therapeutic options in BTC patients is still limited at present, the increased interest in CCA research and the rapidly growing amount of information in the field support a more optimistic horizon in the near future.

## Data Availability

Not applicable.
